# Syndromic Surveillance Using Structured Telehealth Data: Case Study of the First Wave of COVID-19 in Brazil

**DOI:** 10.2196/40036

**Published:** 2023-01-24

**Authors:** Viviane S Boaventura, Malú Grave, Thiago Cerqueira-Silva, Roberto Carreiro, Adélia Pinheiro, Alvaro Coutinho, Manoel Barral Netto

**Affiliations:** 1 Laboratório de Doenças Infecciosas Transmitidas por Vetores Instituto Gonçalo Moniz, Fundação Oswaldo Cruz Salvador Brazil; 2 Faculdade de Medicina Federal University of Bahia Salvador Brazil; 3 Department of Civil Engineering Federal University of Rio de Janeiro Rio de Janeiro Brazil; 4 Fundação Oswaldo Cruz Salvador Brazil; 5 Department of Civil Engineering Fluminense Federal University Niterói Brazil; 6 Instituto Gonçalo Moniz, Fundação Oswaldo Cruz Salvador Brazil; 7 Centre for Data and Knowledge Integration for Health Instituto Gonçalo Moniz, Fundação Oswaldo Cruz Salvador Brazil; 8 Departamento de Ciências da Saúde Universidade Estadual de Santa Cruz Salvador Brazil

**Keywords:** telehealth, telemedicine, disease surveillance, mathematical model, COVID-19, prediction, cases, detection, monitoring, surveillance, computational modeling, spread, transmission, disease, infectious diseases, syndromic

## Abstract

**Background:**

Telehealth has been widely used for new case detection and telemonitoring during the COVID-19 pandemic. It safely provides access to health care services and expands assistance to remote, rural areas and underserved communities in situations of shortage of specialized health professionals. Qualified data are systematically collected by health care workers containing information on suspected cases and can be used as a proxy of disease spread for surveillance purposes. However, the use of this approach for syndromic surveillance has yet to be explored. Besides, the mathematical modeling of epidemics is a well-established field that has been successfully used for tracking the spread of SARS-CoV-2 infection, supporting the decision-making process on diverse aspects of public health response to the COVID-19 pandemic. The response of the current models depends on the quality of input data, particularly the transmission rate, initial conditions, and other parameters present in compartmental models. Telehealth systems may feed numerical models developed to model virus spread in a specific region.

**Objective:**

Herein, we evaluated whether a high-quality data set obtained from a state-based telehealth service could be used to forecast the geographical spread of new cases of COVID-19 and to feed computational models of disease spread.

**Methods:**

We analyzed structured data obtained from a statewide toll-free telehealth service during 4 months following the first notification of COVID-19 in the Bahia state, Brazil. Structured data were collected during teletriage by a health team of medical students supervised by physicians. Data were registered in a responsive web application for planning and surveillance purposes. The data set was designed to quickly identify users, city, residence neighborhood, date, sex, age, and COVID-19–like symptoms. We performed a temporal-spatial comparison of calls reporting COVID-19–like symptoms and notification of COVID-19 cases. The number of calls was used as a proxy of exposed individuals to feed a mathematical model called “susceptible, exposed, infected, recovered, deceased.”

**Results:**

For 181 (43%) out of 417 municipalities of Bahia, the first call to the telehealth service reporting COVID-19–like symptoms preceded the first notification of the disease. The calls preceded, on average, 30 days of the notification of COVID-19 in the municipalities of the state of Bahia, Brazil. Additionally, data obtained by the telehealth service were used to effectively reproduce the spread of COVID-19 in Salvador, the capital of the state, using the “susceptible, exposed, infected, recovered, deceased” model to simulate the spatiotemporal spread of the disease.

**Conclusions:**

Data from telehealth services confer high effectiveness in anticipating new waves of COVID-19 and may help understand the epidemic dynamics.

## Introduction

Telehealth encompasses the distinct ways of interaction between patients and their health care providers. The growing popularity of virtual visits stems from the possibility of faster contact, elimination of transportation time [1], and by providing initial care in remote areas without adequate health care [2], making health care more efficient. Telehealth services can be used for diagnosis, treatment, follow-up, and screening purposes [3]. During the COVID-19 pandemic, telehealth services have been widely used for screening suspected cases [4,5], as limited physical contact reduces everyone’s exposure to COVID-19. This successful strategy safely provides access to health care services and expands assistance to remote rural areas and underserved communities in situations of shortage of specialized health professionals [3]. Using health information platforms, health care workers can systematically collect qualified data on suspected cases. Such data can be used as a proxy for the spread of infectious diseases for health surveillance [6-11].

The mathematical modeling of epidemics is a well-established field that has been successfully used for tracking the spread of SARS-CoV-2 infection, supporting the decision-making process on diverse aspects of public health response to the COVID-19 pandemic. Such models are usually defined as compartmental models. The population under study is divided into compartments based on qualitative characteristics, with different assumptions about the nature and rate of transfer across compartments. The urgency of the COVID-19 pandemic has motivated the need for more research in this area, with several models for this pandemic outbreak being presented in the last few years [12-18]. The response of the current models depends on the quality of input data, particularly the transmission rate, initial conditions, and other parameters present in compartmental models. Telehealth systems may feed numerical models developed to model virus spread in a specific region.

Here, we report using a high-quality data set obtained from a state-based telehealth service for disease surveillance both for forecasting the geographical spread of new cases of COVID-19 and feeding an advanced computational model that tries to reproduce the virus spread dynamics. We used data from a toll-free telehealth service of the 14.8 million population from the state of Bahia, Brazil [19], to simulate the spatiotemporal spread of COVID-19 using a compartmental model with diffusion in Salvador, the state capital.

## Methods

### Telehealth

#### Service and Data Collection

Telecoronavirus was a toll-free phone-teletriage service offered to the population of the state of Bahia, Brazil (population of 14.8 million, 417 municipalities, located in the northeast of the country; Multimedia Appendix 1) [19]. Risk screening was provided for cases suspected of COVID-19 from March 24, 2020, to July 31, 2020, during the first epidemic wave, starting 18 days after the first confirmed case of COVID-19 in the state. This toll-free service operated 7 days a week and was publicized statewide by the state government through television, the internet, social media, billboards, radio, and newspapers. In total, 77% (320/417) of all cities in Bahia and all sanitary districts of the state capital, Salvador, accessed the telehealth service.

Structured data were collected during teletriage by a health team of medical students supervised by physicians. Data were registered in a responsive web application for planning and surveillance purposes. The data set was designed to quickly identify users, city, residence neighborhood, date, sex, age, and COVID-19–like symptoms (fever, cough, breathlessness, rhinorrhea, and gustatory or olfactory disorder). For this study, the call reporting at least one of those symptoms was considered a suspected case of COVID-19.

Strategies to improve the service quality were applied, including educational and technical support for the health care team, updating the online application based on clinical protocols, and monitoring the quality of information registered by the health care team, as previously described [19]. Data of telehealth service were categorized according to the phone call date, the type of COVID-19–like symptom reported by the user, and the city of residence. For Salvador, the state capital, calls were categorized according to the COVID-19–like symptoms and the user’s health district. The COVID-19 cases’ notification date and their related geolocation in the state of Bahia were obtained through the Brazilian Ministry of Health. For Salvador, notification data were obtained through the Salvador Municipal Health Department. We further quantified the cities of the state of Bahia and the districts of Salvador, where phone calls reporting symptoms of COVID-19 preceded the first notification of the disease. The lag in days between the first call and the first notification dates was also annotated.

### Ethical Considerations

Anonymized consolidated data were provided by the Bahia State Health Secretary for research purposes. The ethics committee of the School of Medicine of Bahia approved the project (approval number 4459774, on 12/13/2020). The requirement for obtaining informed consent was waived due to the characteristics of the research.

### Data Source and Preparation for Study

We extracted Telecoronavirus data for Salvador inhabitants (population: 2.88 million; surface area: 692.818 km²) for use in the prediction model. The call records were grouped in epidemiological weeks and residential areas informed by Telecoronavirus users. Additionally, they were grouped in sanitary districts using the list of neighborhoods comprising each of the 13 sanitary districts of the city of Salvador. Official numbers of COVID-19 confirmed cases were also obtained by sanitary districts.

### Use of Telehealth Data for Feeding Epidemiological Modeling

We further tested the hypothesis that telehealth systems can help feed numerical models developed to predict a virus spread in a specific region using a spatiotemporal model presented in [12,20-22] and explained as follows.

#### The Susceptible, Exposed, Infected, Recovered, Deceased Model for COVID-19

The COVID-19 dynamics may be modeled as compartmental models, in which the population under study is divided into compartments and has assumptions about the nature and time rate of transfer from one compartment to another [23]. These models have been used extensively in biological, ecological, and chemical applications [24-26]. They allow for an understanding of the processes at work and predict the dynamics of the epidemic. One of the simplest compartmental models is the “susceptible, infected, removed” model proposed in 1927 by Kermack and McKendrick [27], in which the population is divided into susceptible, infected, and recovered compartments. This basic “susceptible, infected, removed” model can be extended in several ways by enriching the number of compartments, as the “susceptible, exposed, infectious, recovered, deceased” model. Here, we work with a spatiotemporal “susceptible, exposed, infectious, recovered, deceased” model, presented in [20-22], given by,




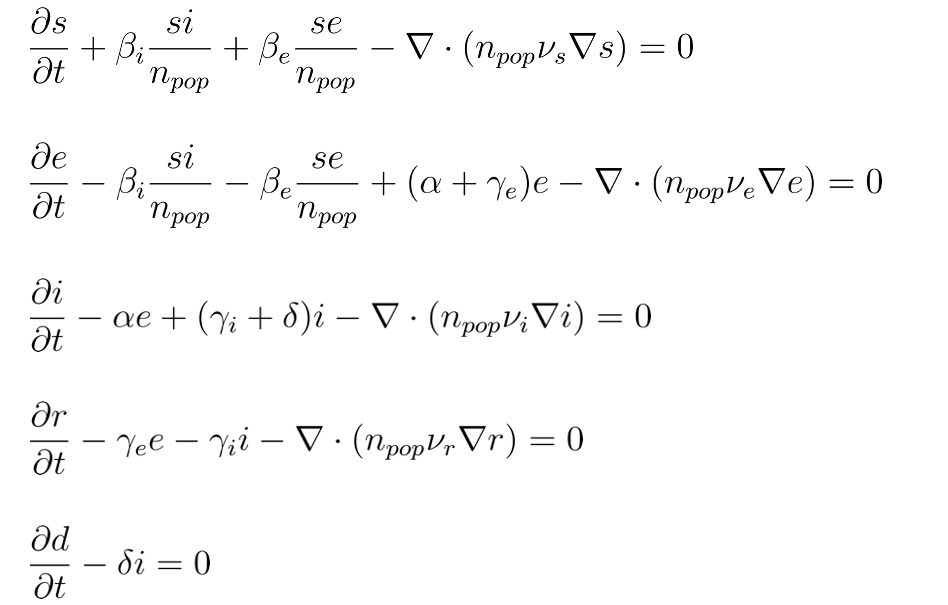




Where *s(x, t)*, *e(x, t)*, *i(x, t)*, *r(x, t)*, and *d(x, t)* denote the densities of the *susceptible*, *exposed*, *infected*, *recovered*, and *deceased* populations, respectively. The sum of all the compartments, except for *d(x, t)*, is represented by *n_pop_*, which is the total living population. β_i_ and β_e_ denote the transmission rates between symptomatic and susceptible individuals and asymptomatic and susceptible individuals, respectively (units 1/days), *a* denotes the incubation period (units 1/days), *γ_e_* corresponds to the asymptomatic recovery rate (units 1/days), *γ_i_* the symptomatic recovery rate (units 1/days), *δ* represents the mortality rate (units 1/days), and *ν_s_, ν_e_, ν_i_, ν_r_* are the diffusion parameters of the different population groups as denoted by the subscripted letters: units km²/(persons.days).

To use this model, we need to define all parameters that govern the system of equations and the initial population of each compartment. Note that all these parameters can be considered time- and space-dependent. We need to infer several hypotheses about the initial conditions, especially those related to the exposed compartment, consisting of asymptomatic cases, which are more challenging to estimate.

#### Model Construction for Salvador, Bahia

We define the beginning of the simulation as April 1, 2020, and simulate 180 days. The initial infected population is set according to the 7 days moving average data provided by the Brazilian Ministry of Health, following the procedure in [12] and [28]. The susceptible population is based on the estimation of the population of each Sanitary district, given by the Brazilian Institute for Geography and Statistics. Recovered and deceased populations start with zero assigned cases (ie, we consider that nobody died or recovered from COVID-19 at the beginning of the simulation).

The exposed compartment is the most nontrivial compartment to assign an initial condition. In previous works, the estimation was based on the amount of the infected population. It was considered that the exposed population could be about 10 times the number of the infected [29]. Here, we consider the 7-day moving average data from telehealth on April 1, 2020, as the initial exposed population. The telehealth data indicated rises in cases approximately 2 weeks before the notification system. Therefore, the calls may indicate a better estimation than the relation with the infected, especially at the epidemic’s beginning.

Each compartment’s population is divided by the area of each sanitary district and distributed in the 12 areas as people/km². Table 1 shows the differences between how the initial exposed population is considered in this work (related to the telehealth system) and how it would be represented when using the old approach (by multiplying the number of infected individuals by 10). Without the need for any calculation, it is possible to see that there is no correlation between the numbers of each approach.

The biological parameters of the simulation are defined based on the literature, as *α = 1/7 day^–1^, γ_i_ = 1/24 day^–1^, γ_e_ = 1/6 day^–1^, δ = 1/160 day^–1^* [21]. On the other hand, the contact rate and the diffusion coefficient have to be estimated. They are based on the social distancing estimation, representing the homestay rate for Bahia.

**Table 1:** Initial exposed population (people/km2) based on 10 times the number of infected and the telehealth calls.

Sanitary district	Ten times the number of infected	Telehealth
Barra Rio Vermelho	27.14	53.00
Brotas	8.57	34.57
Centro Histórico	2.86	13.42
Liberdade	4.29	17.70
Boca do Rio	5.71	23.42
Cabula/Beiru	8.57	43.57
Itapuã	10.00	36.14
Pau da Lima	4.29	25.85
Subúrbio Ferroviário	7.14	30.28
Itapagipe	2.86	18.42
São Caetano or Valéria	7.14	26.57
Cajazeiras	2.86	10.28

## Results

**Figure 1:**
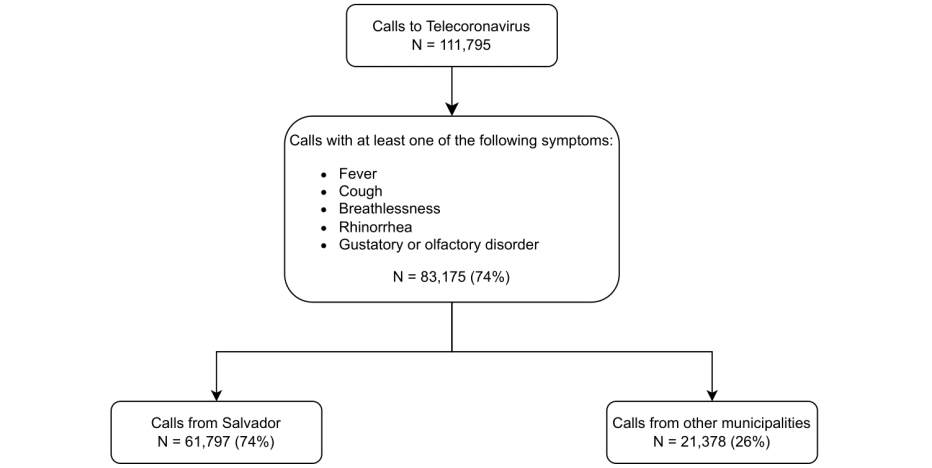
Flowchart of calls for Telecoronavirus service according to local and presence of COVID-19–like symptoms.

The telehealth service received a total of 111,795 calls, 83,175 (74%) of which reporting at least one COVID-19 symptom (fever, cough, breathlessness, rhinorrhea, and gustatory or olfactory disorder) during the first 4 months of the COVID-19 epidemic in the state of Bahia (Figure 1). Olfactory or gustatory dysfunction (smell and taste change), considered highly specific for COVID-19 during the first wave of the pandemic [30-32], was reported in 47% of the calls, suggesting a high frequency of COVID-19 cases among users of the telehealth service.

Calls were registered by 320 (77%) out of 417 cities of Bahia State. The majority of these users of the telehealth service were female (48,873, 60%), with a median age of 38 (IQR 28-49) years. The demand for the service progressively increased between the first and ninth week, with a peak in the 23rd week. In the 4th month of operation, we observed a reduction in the number of daily calls to values close to those at the beginning of the service (Table 2).

**Figure 2:**
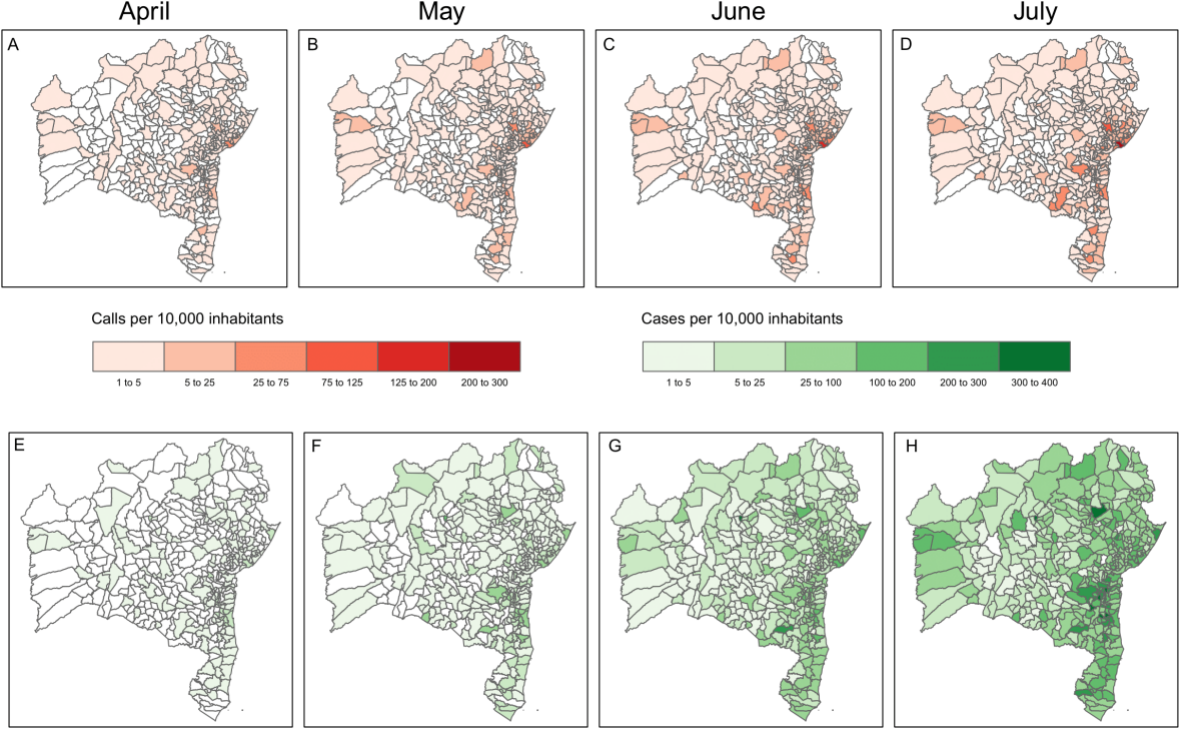
Spatial distribution of calls related to COVID-19–like symptoms (orange) and notifications (green) by months of operation of the Telecoronavirus in Bahia state.

During the first month of operation (April 2020), calls reporting COVID-19–like symptoms were registered in 205 cities, including areas from the north and east of the state. By this time, COVID-19 cases were notified in 136 cities (Figure 2). The first call to the telehealth service preceded the first COVID-19 notification in 181 (43%) out of 417 municipalities of Bahia. In these municipalities, the call occurred on average 30 (IQR 11-42) days before the notification. Additionally, for 68 (38%) of these 181 municipalities, the symptoms registered in the telehealth service included olfactory and gustatory disorders. These symptoms specific for COVID-19 were reported on average 14 (IQR 6-31) days before the first notified COVID-19 case (Multimedia Appendix 2).

**Table 2:** Symptoms reported by users and the number of monthly calls.

Characteristic			Salvador (n=61,797)		Other cities (n=21,378)		State (n=83,175)
Age (years), median (IQR)			39 (29, 50)		37 (27, 48)		38 (28, 49)
**Month, n (%)**							
	March	178 (0.3)		49 (0.2)		227 (0.3)	
	April	10,009 (16)		3464 (16)		13,473 (16)	
	May	23,227 (38)		4822 (23)		28,049 (34)	
	June	20,743 (34)		8634 (40)		29,377 (35)	
	July	7640 (12)		4409 (21)		12,049 (14)	
Sex: female, n (%)			36,315 (60)		12,558 (60)		48,873 (60)
Fever, n (%)			30,501 (49)		10,468 (49)		40,969 (49)
Cough, n (%)			30,307 (49)		11,287 (53)		41,594 (50)
Shortness of breath, n (%)			15,518 (25)		6054 (28)		21,572 (26)
Smell or taste change, n (%)			28,741 (47)		9248 (44)		37,989 (47)

**Figure 3:**
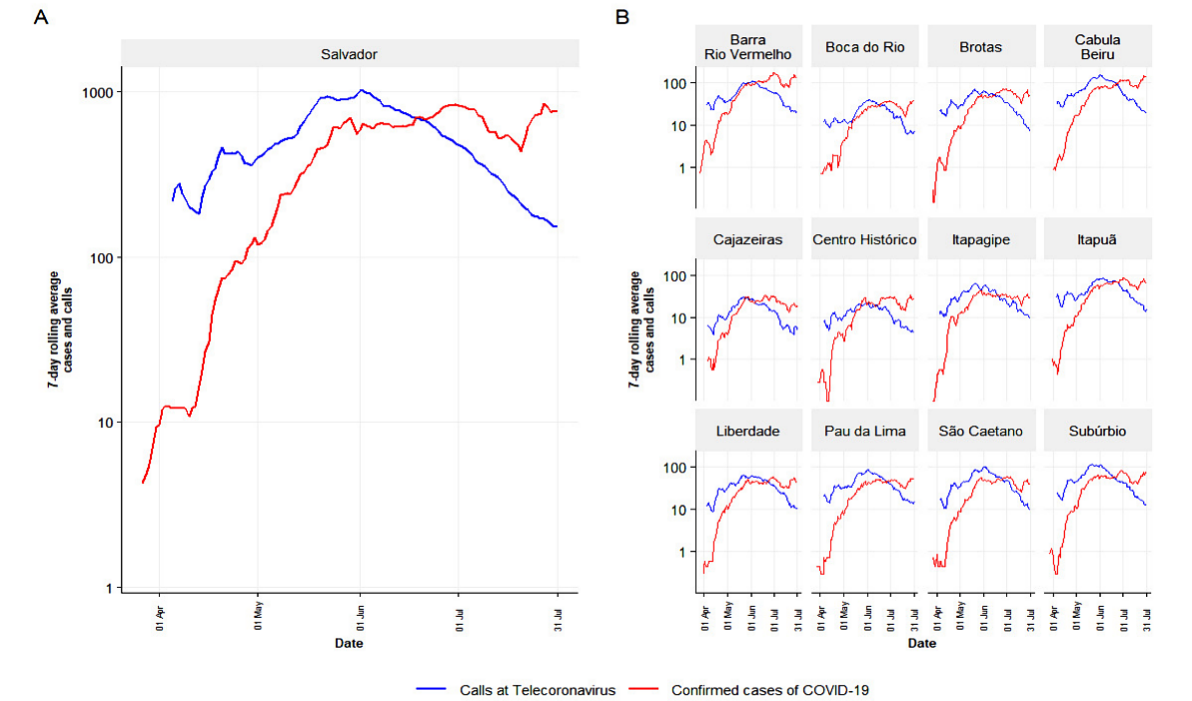
Seven-day rolling average of number of calls reporting COVID-19–like symptoms (blue line) and number of confirmed cases of COVID-19 (red line) in the period of operation of the telehealth service in Salvador, Bahia state, Brazil. (A) All cities. (B) Stratified by sanitary district.

We also analyzed the spatial-temporal distribution of calls and notified cases in the districts of Salvador, the state capital. In all districts, an increase in the number of calls reporting COVID-19–like symptoms preceded an elevation in the number of notifications of COVID-19 (Figure 3).

**Figure 4:**
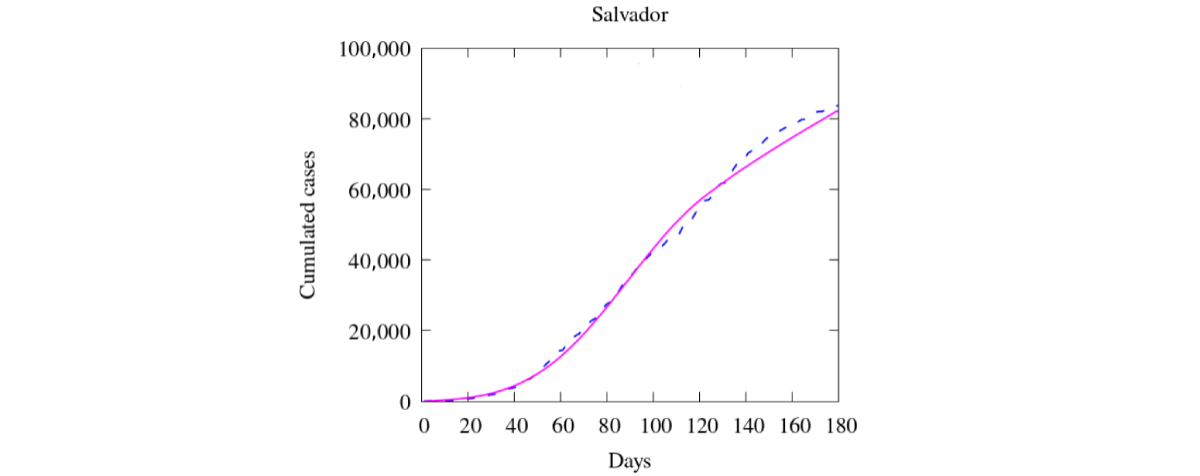
Comparison between simulation and real data of cases at Salvador (total). Dashed blue lines: real data. Pink line: simulation with exposed population based on telehealth calls.

Next, we evaluated if the data obtained with the telehealth service could be helpful in feeding a mathematical model to predict disease spread in Salvador. We simulated 180 days of the epidemic. To validate the results of our model with the available data, we compared the number of notified COVID-19 cases. First, we showed the values obtained for the whole city (Figure 4). Then, we integrated the values of the sanitary districts and plotted them in time. The simulation provides curves of accumulated infection similar to the data of notified cases.

## Discussion

### Principal Findings

A critical challenge in predicting the spread of cases, especially in locals with a low test rate, is to timely estimate the number of suspected cases. Here, we demonstrated that curated data obtained from a telehealth service could be helpful in anticipating areas with new cases of COVID-19. A rise in calls referring to COVID-19–like illness preceded, in about two weeks, the increase in the notification of COVID-19 in the majority of Bahia state cities demonstrating the potential utility of this instrument for syndromic surveillance in the early phase of the epidemic curve. Additionally, it contains relevant information about the disease under investigation, such as the profile of the affected population, type, and frequency of symptoms, which can help characterize susceptible populations, which is important in the context of epidemics caused by new infectious agents. Calls captured in the telehealth service were also valuable in feeding a numerical disease spread model. Data obtained by a toll-free telehealth service can achieve the goals required for surveillance purposes, such as the following: (1) to be collected early after disease onset, (2) to represent the majority of the population in the covered area, and (3) to be rapidly accessed by the decision-making officials [5,33].

First, in an outbreak caused by a pathogen of airborne transmission, such as COVID-19, the fear of contamination reduces seeking face-to-face medical assistance. The majority of individuals with mild symptoms avoid being exposed in clinical care facilities, reducing the number of individuals who get tested, and therefore confirmed infections may be underestimated. From the patient perspective, telehealth assistance becomes more attractive. Accordingly, a substantial increase in telemedicine service was detected in the first weeks of the pandemic [5,34]. Consequently, data from telehealth services become more suitable for surveillance purposes than laboratory-confirmed infection or electronic medical records obtained from health care units.

Second, telehealth services can cover a population from a wide geographic area. The service offered via telephone dismisses reading or writing skills, digital literacy, or internet access and can be used by all age groups and social strata. If the service is toll-free, most of the population may be covered, which is especially important for low- to middle-income countries, as observed in this case.

Third, data sets containing time-sensitive information collected from telehealth services can be accessed simultaneously by public health authorities for syndromic surveillance. Automating data extraction and real-time interpretation of information in the context of relevant public health emergencies is still challenging. Structured surveys designed for telescreening assistance, as described here, can serve this purpose and offer daily reports for the stakeholders. Although several aspects, such as technical or operational, ethical, and legal points, should be addressed to use these data for disease surveillance, analysis of real-time data obtained from telehealth services is feasible. It has already been performed for contact tracing, screening, and monitoring clinical conditions in emergency response to epidemics [35] and can support decision-making on public health policy.

Our result suggests that the data set from a telehealth service may also be helpful as input to a mathematical model to predict COVID-19 spread. The initial conditions of the exposed and infected populations are essential to short-term predictions. The estimate of exposure may be challenging since this population has no symptoms. Therefore, the hypothesis of assuming the number of exposed as the number of calls might be better than an estimation based on the number of infected, since the telehealth data indicated rises in cases approximately 2 weeks before the notification system.

The simulation for a city of about 2.9 million inhabitants showed that most sanitary districts had a good agreement between the actual data and the simulation. The simulation overestimated the cases in 1 district area and underestimated them in 2 areas. Other districts had an excellent agreement until day 100 and lost accuracy afterward. A loss of accuracy in a fast-changing infectious disease is expected since it is challenging to reproduce long-term predictions. The simulation may still be improved due to several aspects needing to be considered. For example, we used estimates for parameters such as transmission rate and diffusion, and it would be possible to use a machine learning mechanism to find the best ones for this case. Moreover, we define that all sanitary districts would receive the same input parameters, and we know that different regions might have performed differently regarding the population’s behavior and restriction policies. Besides, we used only spatial spread mechanisms due to diffusion and further use of different approaches, as convection and source terms could have offered better estimates. However, the central idea of this report is to show that telehealth data impact a robust algorithm that worked well to simulate the COVID-19 behavior of different regions (Italy, Brazil, and the United States [12]).

Collecting real-time data may help predictors forecast new surges and prepare the population, authorities, and health systems. Moreover, we may infer that our hypothesis works since our simulation with the telehealth data have provided promising results.

### Limitations

Our study has some limitations. We used COVID-19–like symptoms as a proxy for confirmed infection. Although this is not accurate, the number of phone calls and the trend in service assessment may suggest the direction and amount of disease spread early on. It shall also be stressed that confirmatory tests may not be available in the early stages of a new epidemic infection. In such cases, the use of the syndromic approach, as employed here, is a critical element for predicting infection spreading. Another significant limitation is that the telehealth service was not equally publicized in all cities, which may have implications for interpreting surveillance data in some areas.

### Conclusion

In conclusion, data from telehealth services help model COVID-19 spread and may be helpful in other health situations. Telehealth data and digital health technologies for monitoring disease spread may be especially useful considering the resurgence of new SARS-CoV-2 variants [36]. Data collected from primary health care systems can be used for monitoring the dynamics of COVID-19 cases and the geographic localization of cases. Considering the continuous expansion of telehealth and telemedicine tools in the health care system, the availability of such data may prove a critical tool for modern epidemiology.

## Appendix

Multimedia Appendix 1 Brazil’s map showing the state of Bahia and the capital Salvador.

Multimedia Appendix 2 Date of first confirmed case, first call, and first call reporting smell or taste disorder for each municipality of Bahia state, Brazil.

## Data Availability

Our agreement with the Bahia State Health Secretary for accessing the database denies authorization of access to a third party. Request for accessing the database should be addressed to the state government, Secretaria de Saúde do Estado da Bahia.
