# Supplementary figures and images for: Syndromic Surveillance Using Structured Telehealth Data: Case Study of the First Wave of COVID-19 in Brazil
Source: JMIR Public Health Surveill. 2023 Jan 24;9:e40036. doi: 10.2196/40036 (PMC9875555; doi:10.2196/40036)

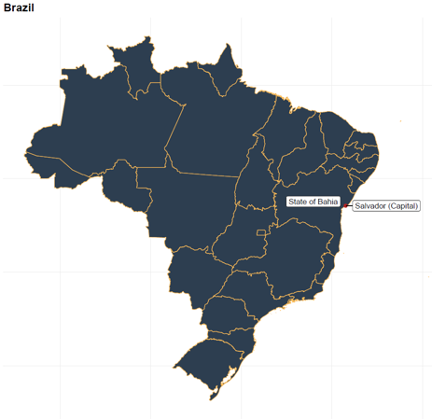

Supplement: Multimedia Appendix 1 [file publichealth_v9i1e40036_app1.png]
