# Supplementary material for: Syndromic Surveillance Using Structured Telehealth Data: Case Study of the First Wave of COVID-19 in Brazil
Source: JMIR Public Health Surveill. 2023 Jan 24;9:e40036. doi: 10.2196/40036 (PMC9875555; doi:10.2196/40036)
Supplement: Multimedia Appendix 2 [file publichealth_v9i1e40036_app2.docx]

**Multimedia Appendix 2.** Date of first confirmed case, first call, and first call reporting smell or taste disorder for each municipality of Bahia state, Brazil.

| **City** | **First Confirmed Case** | **First Call - Telecoronavirus** | **First Call reporting loss of smell or taste** |
| --- | --- | --- | --- |
| Abaíra | 2020-04-13 | NA | NA |
| Abaré | 2020-05-15 | 2020-07-22 | NA |
| Acajutiba | 2020-04-22 | 2020-05-04 | 2020-06-23 |
| Adustina | 2020-04-04 | 2020-04-21 | 2020-04-21 |
| Água Fria | 2020-04-23 | 2020-04-05 | 2020-04-15 |
| Érico Cardoso | 2020-04-16 | 2020-05-12 | 2020-05-12 |
| Aiquara | 2020-03-26 | 2020-04-01 | 2020-04-08 |
| Alagoinhas | 2020-05-30 | 2020-04-20 | 2020-06-03 |
| Alcobaça | 2020-04-23 | 2020-04-20 | NA |
| Almadina | 2020-05-10 | 2020-04-01 | 2020-04-20 |
| Amargosa | 2020-04-16 | 2020-04-05 | 2020-04-11 |
| Amélia Rodrigues | 2020-06-21 | 2020-04-30 | NA |
| América Dourada | 2020-05-14 | 2020-06-16 | 2020-06-27 |
| Anagé | 2020-06-02 | NA | NA |
| Andaraí | 2020-06-13 | 2020-04-15 | NA |
| Andorinha | 2020-06-08 | 2020-04-16 | 2020-06-05 |
| Angical | 2020-05-10 | 2020-06-22 | 2020-07-27 |
| Anguera | 2020-05-31 | NA | NA |
| Antas | 2020-05-25 | 2020-04-01 | NA |
| Antônio Cardoso | 2020-06-14 | 2020-04-05 | 2020-05-04 |
| Antônio Gonçalves | 2020-05-23 | 2020-05-11 | 2020-05-11 |
| Aporá | 2020-05-21 | 2020-06-08 | 2020-06-17 |
| Apuarema | 2020-05-14 | 2020-05-20 | 2020-05-30 |
| Aracatu | 2020-04-28 | 2020-05-10 | 2020-06-02 |
| Araças | 2020-04-08 | 2020-04-03 | 2020-05-18 |
| Araci | 2020-06-02 | 2020-05-27 | NA |
| Aramari | 2020-04-29 | NA | NA |
| Arataca | 2020-05-28 | 2020-05-25 | 2020-06-29 |
| Aratuípe | 2020-04-08 | 2020-04-06 | 2020-04-26 |
| Aurelino Leal | 2020-05-26 | 2020-06-19 | 2020-06-19 |
| Baianópolis | 2020-06-19 | 2020-04-20 | NA |
| Baixa Grande | 2020-05-15 | NA | NA |
| Banzaê | 2020-03-31 | 2020-06-06 | 2020-06-06 |
| Barra | 2020-06-07 | 2020-04-14 | NA |
| Barra da Estiva | 2020-04-06 | 2020-05-29 | NA |
| Barra do Choça | 2020-05-23 | NA | NA |
| Barra do Mendes | 2020-04-01 | 2020-04-29 | 2020-05-13 |
| Barra do Rocha | 2020-03-22 | 2020-03-31 | 2020-04-15 |
| Barreiras | 2020-07-04 | NA | NA |
| Barro Alto | 2020-04-19 | 2020-04-26 | NA |
| Barrocas | 2020-05-10 | 2020-04-15 | 2020-04-15 |
| Governador Lomanto Júnior | 2020-04-02 | 2020-05-22 | 2020-06-14 |
| Belmonte | 2020-06-14 | 2020-07-30 | NA |
| Belo Campo | 2020-05-24 | 2020-06-20 | NA |
| Biritinga | 2020-06-06 | NA | NA |
| Boa Nova | 2020-05-07 | 2020-07-10 | 2020-07-10 |
| Boa Vista do Tupim | 2020-05-15 | 2020-04-14 | 2020-06-01 |
| Bom Jesus da Lapa | 2020-05-25 | NA | NA |
| Bom Jesus da Serra | 2020-06-06 | NA | NA |
| Boninal | 2020-06-04 | 2020-07-17 | NA |
| Bonito | 2020-05-13 | NA | NA |
| Boquira | 2020-06-09 | NA | NA |
| Botuporã | 2020-06-11 | 2020-04-03 | NA |
| Brejões | 2020-08-04 | NA | NA |
| Brejolândia | 2020-06-05 | 2020-05-24 | 2020-07-08 |
| Brotas de Macaúbas | 2020-03-23 | 2020-04-03 | 2020-05-04 |
| Brumado | 2020-04-16 | 2020-04-14 | 2020-04-14 |
| Buerarema | 2020-05-30 | 2020-06-03 | NA |
| Buritirama | 2020-06-13 | NA | NA |
| Caatiba | 2020-05-15 | 2020-05-01 | 2020-05-01 |
| Cabaceiras do Paraguaçu | 2020-04-04 | 2020-04-15 | 2020-04-17 |
| Cachoeira | 2020-05-29 | 2020-04-14 | 2020-04-14 |
| Caculé | 2020-05-18 | NA | NA |
| Caém | 2020-04-26 | NA | NA |
| Caetanos | 2020-05-19 | 2020-04-23 | 2020-06-01 |
| Caetité | 2020-06-27 | 2020-04-18 | NA |
| Cafarnaum | 2020-05-12 | 2020-05-22 | 2020-05-22 |
| Cairu | 2020-04-25 | 2020-07-02 | 2020-07-02 |
| Caldeirão Grande | 2020-04-10 | 2020-04-07 | 2020-04-07 |
| Camacan | 2020-03-20 | 2020-03-31 | 2020-04-04 |
| Camaçari | 2020-04-26 | 2020-04-06 | 2020-04-20 |
| Camamu | 2020-04-23 | 2020-06-11 | NA |
| Campo Alegre de Lourdes | 2020-04-06 | 2020-04-14 | 2020-06-07 |
| Campo Formoso | 2020-08-25 | 2020-05-02 | 2020-05-02 |
| Canápolis | 2020-03-25 | 2020-04-16 | NA |
| Canarana | 2020-04-13 | 2020-04-30 | 2020-07-12 |
| Canavieiras | 2020-06-03 | 2020-07-19 | 2020-07-19 |
| Candeal | 2020-03-31 | 2020-03-31 | 2020-04-14 |
| Candeias | 2020-05-31 | 2020-04-15 | NA |
| Candiba | 2020-05-23 | 2020-04-14 | 2020-04-22 |
| Cândido Sales | 2020-04-10 | 2020-05-08 | 2020-05-19 |
| Cansanção | 2020-05-30 | NA | NA |
| Canudos | 2020-05-10 | 2020-07-01 | NA |
| Capela do Alto Alegre | 2020-04-11 | 2020-05-11 | 2020-05-28 |
| Capim Grosso | 2020-05-26 | 2020-04-14 | NA |
| Caraíbas | 2020-06-04 | 2020-06-29 | NA |
| Caravelas | 2020-05-18 | 2020-04-14 | 2020-04-14 |
| Cardeal da Silva | 2020-06-06 | 2020-04-18 | 2020-06-05 |
| Carinhanha | 2020-05-30 | 2020-04-19 | 2020-06-05 |
| Casa Nova | 2020-04-18 | 2020-04-14 | 2020-04-14 |
| Castro Alves | 2020-06-13 | NA | NA |
| Catolândia | 2020-03-31 | 2020-04-18 | 2020-04-18 |
| Catu | 2020-07-10 | NA | NA |
| Caturama | 2020-05-31 | NA | NA |
| Central | 2020-06-05 | NA | NA |
| Chorrochó | 2020-05-29 | 2020-04-17 | 2020-04-17 |
| Cícero Dantas | 2020-05-24 | 2020-04-06 | NA |
| Cipó | 2020-03-31 | 2020-04-14 | NA |
| Coaraci | 2020-05-19 | NA | NA |
| Cocos | 2020-05-14 | 2020-04-01 | 2020-04-15 |
| Conceição da Feira | 2020-05-15 | 2020-04-04 | NA |
| Conceição do Almeida | 2020-04-08 | 2020-04-13 | 2020-06-10 |
| Conceição do Coité | 2020-03-22 | 2020-04-02 | 2020-04-25 |
| Conceição do Jacuípe | 2020-03-24 | 2020-04-22 | 2020-04-24 |
| Conde | 2020-05-23 | 2020-06-02 | 2020-06-21 |
| Condeúba | 2020-07-12 | NA | NA |
| Contendas do Sincorá | 2020-04-18 | 2020-04-10 | 2020-06-02 |
| Coração de Maria | 2020-06-11 | NA | NA |
| Cordeiros | 2020-06-26 | NA | NA |
| Coribe | 2020-05-28 | 2020-07-30 | NA |
| Coronel João sá | 2020-06-02 | 2020-05-07 | 2020-05-07 |
| Correntina | 2020-06-30 | 2020-05-13 | NA |
| Cotegipe | 2020-04-21 | 2020-04-08 | NA |
| Cravolândia | 2020-05-26 | 2020-06-11 | NA |
| Crisópolis | 2020-05-15 | 2020-05-03 | 2020-05-03 |
| Cristópolis | 2020-04-24 | 2020-04-02 | 2020-04-14 |
| Cruz Das Almas | 2020-04-16 | 2020-04-14 | 2020-04-14 |
| Curaçá | 2020-04-28 | 2020-05-18 | NA |
| Dário Meira | 2020-03-30 | 2020-03-31 | 2020-04-08 |
| Dias D´ávila | 2020-05-10 | 2020-07-02 | 2020-07-02 |
| Dom Basílio | 2020-06-10 | 2020-05-06 | NA |
| Dom Macedo Costa | 2020-06-26 | 2020-06-30 | NA |
| Elísio Medrado | 2020-05-12 | 2020-05-17 | 2020-05-17 |
| Encruzilhada | 2020-05-12 | 2020-04-01 | 2020-04-11 |
| Entre Rios | 2020-06-11 | NA | NA |
| Esplanada | 2020-05-23 | 2020-04-01 | NA |
| Euclides da Cunha | 2020-04-08 | 2020-04-14 | 2020-06-08 |
| Eunápolis | 2020-04-15 | 2020-03-31 | 2020-04-13 |
| Fátima | 2020-05-27 | 2020-06-06 | NA |
| Feira da Mata | 2020-06-27 | 2020-06-13 | 2020-06-13 |
| Feira de Santana | 2020-03-06 | 2020-03-31 | 2020-04-04 |
| Filadélfia | 2020-05-31 | 2020-04-14 | NA |
| Firmino Alves | 2020-06-03 | 2020-04-07 | 2020-04-07 |
| Floresta Azul | 2020-04-14 | 2020-04-16 | 2020-05-10 |
| Formosa do Rio Preto | 2020-06-04 | 2020-04-14 | NA |
| Gandu | 2020-04-04 | 2020-04-15 | 2020-05-25 |
| Gavião | 2020-08-03 | NA | NA |
| Gentio do Ouro | 2020-06-11 | NA | NA |
| Glória | 2020-05-24 | 2020-04-16 | 2020-04-16 |
| Gongogi | 2020-04-08 | NA | NA |
| Governador Mangabeira | 2020-05-02 | 2020-04-16 | 2020-06-19 |
| Guajeru | 2020-06-08 | NA | NA |
| Guanambi | 2020-04-18 | 2020-05-03 | 2020-05-14 |
| Guaratinga | 2020-04-30 | 2020-04-20 | 2020-04-20 |
| Heliópolis | 2020-06-27 | 2020-04-15 | NA |
| Iaçu | 2020-05-15 | 2020-04-03 | 2020-05-18 |
| Ibiassucê | 2020-06-27 | NA | NA |
| Ibicaraí | 2020-04-22 | 2020-04-13 | 2020-06-17 |
| Ibicoara | 2020-06-25 | 2020-04-16 | 2020-04-16 |
| Ibicuí | 2020-06-04 | 2020-04-15 | 2020-07-30 |
| Ibipeba | 2020-05-31 | 2020-06-03 | NA |
| Ibipitanga | 2020-06-05 | 2020-06-08 | 2020-06-08 |
| Ibiquera | 2020-06-27 | NA | NA |
| Ibirapitanga | 2020-05-02 | 2020-05-15 | 2020-06-30 |
| Ibirapuã | 2020-05-23 | 2020-05-23 | 2020-05-23 |
| Ibirataia | 2020-04-05 | 2020-04-25 | 2020-04-25 |
| Ibitiara | 2020-05-15 | NA | NA |
| Ibititá | 2020-07-16 | NA | NA |
| Ibotirama | 2020-04-06 | 2020-04-19 | NA |
| Ichu | 2020-06-27 | 2020-06-25 | 2020-06-25 |
| Igaporã | 2020-06-30 | NA | NA |
| Igrapiúna | 2020-05-15 | 2020-04-15 | NA |
| Iguaí | 2020-05-29 | 2020-04-16 | 2020-05-30 |
| Ilhéus | 2020-03-25 | 2020-03-31 | 2020-04-13 |
| Inhambupe | 2020-05-15 | 2020-05-07 | 2020-05-13 |
| Ipecaetá | 2020-05-15 | 2020-06-23 | NA |
| Ipiaú | 2020-03-27 | 2020-04-01 | 2020-04-08 |
| Ipirá | 2020-04-20 | 2020-04-13 | 2020-05-25 |
| Ipupiara | 2020-07-24 | NA | NA |
| Irajuba | 2020-07-05 | NA | NA |
| Iramaia | 2020-06-05 | 2020-06-10 | 2020-06-10 |
| Iraquara | 2020-06-23 | NA | NA |
| Irará | 2020-05-11 | 2020-04-05 | 2020-05-21 |
| Irecê | 2020-04-15 | 2020-04-13 | 2020-04-19 |
| Itabela | 2020-04-20 | 2020-05-14 | 2020-05-14 |
| Itaberaba | 2020-04-20 | 2020-04-15 | 2020-04-27 |
| Itabuna | 2020-03-19 | 2020-03-31 | 2020-04-07 |
| Itacaré | 2020-04-25 | 2020-04-14 | 2020-05-23 |
| Itaeté | 2020-05-22 | 2020-05-21 | NA |
| Itagi | 2020-04-08 | 2020-05-04 | NA |
| Itagibá | 2020-03-28 | 2020-06-10 | 2020-06-30 |
| Itagimirim | 2020-06-29 | 2020-06-18 | NA |
| Itaguaçu da Bahia | 2020-07-01 | 2020-04-22 | NA |
| Itaju do Colônia | 2020-06-23 | 2020-06-20 | 2020-06-20 |
| Itajuípe | 2020-03-31 | 2020-04-14 | 2020-05-16 |
| Itamaraju | 2020-03-29 | 2020-04-03 | 2020-04-04 |
| Itamari | 2020-04-20 | 2020-05-03 | 2020-06-28 |
| Itambé | 2020-06-14 | 2020-05-27 | NA |
| Itanagra | 2020-05-21 | NA | NA |
| Itanhém | 2020-05-23 | 2020-06-03 | NA |
| Itaparica | 2020-04-26 | 2020-04-04 | 2020-04-30 |
| Itapé | 2020-04-13 | 2020-04-14 | 2020-06-06 |
| Itapebi | 2020-04-09 | 2020-07-01 | 2020-07-01 |
| Itapetinga | 2020-04-04 | 2020-04-02 | 2020-04-14 |
| Itapicuru | 2020-05-10 | 2020-05-07 | NA |
| Itapitanga | 2020-05-09 | NA | NA |
| Itaquara | 2020-05-30 | NA | NA |
| Itarantim | 2020-04-05 | 2020-04-14 | 2020-06-15 |
| Itatim | 2020-04-08 | 2020-04-21 | 2020-04-21 |
| Itiruçu | 2020-06-10 | 2020-05-06 | 2020-06-19 |
| Itiúba | 2020-05-18 | 2020-04-30 | NA |
| Itororó | 2020-03-29 | 2020-04-20 | 2020-06-22 |
| Ituaçu | 2020-06-01 | NA | NA |
| Ituberá | 2020-04-09 | 2020-04-14 | 2020-06-02 |
| Iuiú | 2020-06-06 | NA | NA |
| Jaborandi | 2020-05-23 | 2020-05-10 | 2020-05-10 |
| Jacaraci | 2020-05-23 | NA | NA |
| Jacobina | 2020-05-06 | 2020-04-01 | 2020-04-15 |
| Jaguaquara | 2020-04-10 | 2020-04-01 | 2020-05-12 |
| Jaguarari | 2020-04-28 | 2020-04-29 | NA |
| Jaguaripe | 2020-05-22 | 2020-04-07 | 2020-06-21 |
| Jandaíra | 2020-06-02 | 2020-06-20 | 2020-06-20 |
| Jequié | 2020-03-23 | 2020-03-31 | 2020-04-06 |
| Jeremoabo | 2020-06-10 | 2020-04-02 | 2020-06-15 |
| Jiquiriçá | 2020-05-24 | 2020-04-22 | NA |
| Jitaúna | 2020-05-06 | 2020-04-21 | NA |
| João Dourado | 2020-05-28 | 2020-06-04 | NA |
| Juazeiro | 2020-03-23 | 2020-03-31 | 2020-04-13 |
| Jucuruçu | 2020-06-28 | NA | NA |
| Jussara | 2020-06-05 | 2020-06-25 | NA |
| Jussari | 2020-05-01 | 2020-04-19 | NA |
| Jussiape | 2020-05-03 | NA | NA |
| Lafaiete Coutinho | 2020-05-12 | NA | NA |
| Lagoa Real | 2020-07-15 | NA | NA |
| Laje | 2020-04-25 | 2020-04-30 | 2020-07-06 |
| Lajedão | 2020-05-23 | NA | NA |
| Lajedinho | 2020-07-08 | NA | NA |
| Lajedo do Tabocal | 2020-04-26 | NA | NA |
| Lamarão | 2020-05-23 | 2020-04-07 | NA |
| Lapão | 2020-05-22 | 2020-05-21 | 2020-05-21 |
| Lauro de Freitas | 2020-03-19 | 2020-03-31 | 2020-04-04 |
| Lençóis | 2020-06-27 | 2020-06-01 | NA |
| Licínio de Almeida | 2020-04-23 | NA | NA |
| Livramento de Nossa Senhora | 2020-04-25 | 2020-04-14 | 2020-06-03 |
| Luís Eduardo Magalhães | 2020-04-11 | 2020-04-13 | 2020-04-14 |
| Macajuba | 2020-06-17 | 2020-05-08 | NA |
| Macarani | 2020-05-26 | 2020-04-19 | 2020-04-20 |
| Macaúbas | 2020-06-01 | 2020-07-14 | 2020-07-14 |
| Macururé | 2020-06-27 | NA | NA |
| Madre de Deus | 2020-05-05 | 2020-04-13 | 2020-05-11 |
| Maetinga | 2020-06-03 | NA | NA |
| Maiquinique | 2020-05-29 | NA | NA |
| Mairi | 2020-05-21 | 2020-06-02 | 2020-06-02 |
| Malhada | 2020-06-07 | 2020-04-28 | NA |
| Malhada de Pedras | 2020-06-01 | NA | NA |
| Manoel Vitorino | 2020-05-10 | 2020-04-29 | 2020-04-29 |
| Mansidão | 2020-05-31 | NA | NA |
| Maracás | 2020-04-29 | 2020-04-06 | 2020-04-19 |
| Maragogipe | 2020-04-24 | 2020-04-01 | 2020-05-20 |
| Maraú | 2020-04-29 | 2020-04-19 | 2020-04-19 |
| Marcionílio Souza | 2020-05-25 | NA | NA |
| Mascote | 2020-05-23 | NA | NA |
| Mata de São João | 2020-04-28 | 2020-03-31 | 2020-04-04 |
| Matina | 2020-06-04 | 2020-04-18 | NA |
| Medeiros Neto | 2020-03-31 | 2020-04-19 | 2020-06-07 |
| Miguel Calmon | 2020-05-15 | 2020-05-13 | NA |
| Milagres | 2020-05-30 | 2020-04-08 | 2020-04-08 |
| Mirangaba | 2020-05-15 | NA | NA |
| Mirante | 2020-04-18 | 2020-04-15 | 2020-04-15 |
| Monte Santo | 2020-05-16 | 2020-04-16 | 2020-05-28 |
| Morpará | 2020-04-20 | 2020-06-04 | 2020-06-16 |
| Morro do Chapéu | 2020-05-13 | 2020-04-15 | 2020-05-14 |
| Mortugaba | 2020-06-07 | 2020-04-23 | NA |
| Mucugê | 2020-04-20 | NA | NA |
| Mucuri | 2020-05-14 | 2020-05-05 | 2020-06-01 |
| Mulungu do Morro | 2020-05-31 | NA | NA |
| Mundo Novo | 2020-05-14 | 2020-07-25 | NA |
| Muniz Ferreira | 2020-05-30 | 2020-06-14 | 2020-06-14 |
| Muquém de São Francisco | 2020-06-06 | NA | NA |
| Muritiba | 2020-05-14 | 2020-04-06 | 2020-04-14 |
| Mutuípe | 2020-05-18 | 2020-05-23 | NA |
| Nazaré | 2020-05-03 | 2020-04-26 | 2020-05-16 |
| Nilo Peçanha | 2020-04-25 | 2020-07-20 | 2020-07-20 |
| Nordestina | 2020-05-02 | NA | NA |
| Nova Canaã | 2020-06-07 | 2020-04-20 | NA |
| Nova Fátima | 2020-05-24 | 2020-06-01 | 2020-06-03 |
| Nova Ibiá | 2020-05-15 | 2020-06-16 | 2020-06-16 |
| Nova Itarana | 2020-06-26 | 2020-04-06 | NA |
| Nova Redenção | 2020-06-15 | 2020-03-31 | NA |
| Nova Soure | 2020-03-30 | 2020-04-14 | 2020-07-06 |
| Nova Viçosa | 2020-05-07 | 2020-04-21 | 2020-06-16 |
| Novo Horizonte | 2020-06-27 | NA | NA |
| Novo Triunfo | 2020-05-29 | NA | NA |
| Olindina | 2020-05-23 | 2020-04-20 | 2020-06-24 |
| Oliveira Dos Brejinhos | 2020-04-25 | 2020-05-08 | 2020-05-22 |
| Ouriçangas | 2020-04-05 | 2020-05-10 | NA |
| Ourolândia | 2020-05-30 | NA | NA |
| Palmas de Monte Alto | 2020-06-03 | 2020-05-25 | 2020-05-25 |
| Palmeiras | 2020-04-05 | 2020-05-15 | NA |
| Paramirim | 2020-04-15 | 2020-04-03 | 2020-06-01 |
| Paratinga | 2020-05-23 | 2020-04-23 | NA |
| Paripiranga | 2020-05-30 | NA | NA |
| Pau Brasil | 2020-05-02 | NA | NA |
| Paulo Afonso | 2020-04-21 | 2020-04-01 | 2020-04-07 |
| pé de Serra | 2020-06-05 | 2020-04-14 | 2020-06-10 |
| Pedrão | 2020-05-31 | 2020-06-10 | NA |
| Pedro Alexandre | 2020-05-30 | 2020-04-29 | 2020-06-17 |
| Piatã | 2020-05-15 | NA | NA |
| Pilão Arcado | 2020-05-03 | 2020-04-03 | NA |
| Pindaí | 2020-05-31 | NA | NA |
| Pindobaçu | 2020-05-31 | 2020-07-08 | NA |
| Pintadas | 2020-05-26 | NA | NA |
| Piraí do Norte | 2020-05-28 | NA | NA |
| Piripá | 2020-04-05 | NA | NA |
| Piritiba | 2020-05-18 | 2020-05-11 | NA |
| Planaltino | 2020-07-19 | 2020-04-29 | NA |
| Planalto | 2020-06-05 | 2020-06-27 | 2020-06-27 |
| Poções | 2020-05-30 | 2020-04-14 | 2020-06-05 |
| Pojuca | 2020-03-29 | 2020-04-05 | 2020-04-22 |
| Ponto Novo | 2020-05-15 | 2020-05-25 | 2020-07-13 |
| Porto Seguro | 2020-03-16 | 2020-04-02 | 2020-04-10 |
| Potiraguá | 2020-05-09 | 2020-07-17 | NA |
| Prado | 2020-03-17 | 2020-04-16 | 2020-06-06 |
| Presidente Dutra | 2020-05-10 | 2020-06-25 | NA |
| Presidente Jânio Quadros | 2020-05-15 | 2020-04-06 | 2020-07-14 |
| Presidente Tancredo Neves | 2020-05-10 | 2020-04-07 | 2020-05-19 |
| Queimadas | 2020-05-14 | 2020-05-15 | 2020-05-21 |
| Quijingue | 2020-06-07 | 2020-07-15 | 2020-07-15 |
| Quixabeira | 2020-05-12 | 2020-04-07 | NA |
| Rafael Jambeiro | 2020-05-02 | 2020-05-12 | NA |
| Remanso | 2020-05-04 | 2020-04-14 | 2020-05-08 |
| Retirolândia | 2020-05-03 | 2020-04-15 | 2020-04-25 |
| Riachão Das Neves | 2020-05-26 | NA | NA |
| Riachão do Jacuípe | 2020-05-19 | 2020-04-14 | 2020-05-27 |
| Riacho de Santana | 2020-05-23 | NA | NA |
| Ribeira do Amparo | 2020-05-23 | NA | NA |
| Ribeira do Pombal | 2020-04-19 | 2020-04-26 | 2020-06-18 |
| Ribeirão do Largo | 2020-06-02 | 2020-04-14 | 2020-04-14 |
| Rio de Contas | 2020-07-04 | NA | NA |
| Rio do Antônio | 2020-05-27 | 2020-04-19 | NA |
| Rio do Pires | 2020-04-10 | 2020-04-16 | NA |
| Rio Real | 2020-04-22 | 2020-04-21 | 2020-04-21 |
| Rodelas | 2020-05-12 | NA | NA |
| Ruy Barbosa | 2020-05-13 | 2020-04-14 | 2020-04-23 |
| Salinas da Margarida | 2020-05-27 | 2020-04-01 | 2020-05-29 |
| Salvador | 2020-03-13 | 2020-03-30 | 2020-04-04 |
| Santa Bárbara | 2020-04-28 | 2020-04-14 | 2020-05-17 |
| Santa Brígida | 2020-05-24 | NA | NA |
| Santa Cruz Cabrália | 2020-04-02 | 2020-04-19 | 2020-04-19 |
| Santa Cruz da Vitória | 2020-05-31 | 2020-06-18 | 2020-06-18 |
| Santa Inês | 2020-05-26 | 2020-04-14 | 2020-07-11 |
| Santaluz | 2020-04-09 | 2020-04-14 | 2020-04-30 |
| Santa Luzia | 2020-04-05 | 2020-04-23 | 2020-07-07 |
| Santa Maria da Vitória | 2020-05-29 | 2020-06-01 | 2020-06-06 |
| Santana | 2020-04-14 | 2020-05-13 | 2020-05-13 |
| Santanópolis | 2020-04-27 | 2020-04-25 | NA |
| Santa Rita de Cássia | 2020-06-27 | NA | NA |
| Santa Teresinha | 2020-05-08 | 2020-06-11 | 2020-06-11 |
| Santo Amaro | 2020-04-29 | 2020-03-31 | 2020-04-18 |
| Santo Antônio de Jesus | 2020-05-02 | 2020-04-01 | 2020-04-14 |
| Santo Estêvão | 2020-05-09 | 2020-04-05 | 2020-04-08 |
| São Desidério | 2020-05-14 | 2020-04-14 | 2020-04-15 |
| São Domingos | 2020-03-25 | NA | NA |
| São Félix | 2020-04-28 | 2020-04-14 | 2020-06-11 |
| São Félix do Coribe | 2020-05-29 | 2020-05-02 | 2020-05-02 |
| São Felipe | 2020-06-27 | 2020-06-28 | 2020-06-28 |
| São Francisco do Conde | 2020-04-06 | 2020-04-02 | 2020-05-18 |
| São Gabriel | 2020-06-06 | NA | NA |
| São Gonçalo Dos Campos | 2020-05-02 | 2020-04-01 | 2020-04-15 |
| São José da Vitória | 2020-04-23 | 2020-07-01 | 2020-07-01 |
| São José do Jacuípe | 2020-05-30 | 2020-04-20 | 2020-05-13 |
| São Miguel Das Matas | 2020-06-08 | 2020-04-26 | NA |
| São Sebastião do Passé | 2020-05-05 | 2020-04-03 | 2020-04-10 |
| Sapeaçu | 2020-05-24 | 2020-04-18 | 2020-04-20 |
| Sátiro Dias | 2020-04-24 | 2020-04-13 | NA |
| Saubara | 2020-05-02 | 2020-05-15 | 2020-05-22 |
| Saúde | 2020-05-15 | 2020-04-20 | NA |
| Seabra | 2020-04-28 | 2020-04-23 | 2020-05-14 |
| Sebastião Laranjeiras | 2020-07-04 | NA | NA |
| Senhor do Bonfim | 2020-05-12 | 2020-04-01 | 2020-05-05 |
| Serra do Ramalho | 2020-05-15 | 2020-04-20 | NA |
| Sento sé | 2020-04-12 | 2020-04-20 | NA |
| Serra Dourada | 2020-06-02 | NA | NA |
| Serra Preta | 2020-04-22 | 2020-04-15 | 2020-05-27 |
| Serrinha | 2020-04-08 | 2020-04-01 | 2020-04-07 |
| Serrolândia | 2020-05-07 | 2020-05-03 | 2020-05-03 |
| Simões Filho | 2020-04-04 | 2020-03-31 | 2020-04-04 |
| Sítio do Mato | 2020-08-05 | NA | NA |
| Sítio do Quinto | 2020-06-04 | NA | NA |
| Sobradinho | 2020-05-14 | 2020-04-19 | 2020-05-17 |
| Souto Soares | 2020-05-25 | NA | NA |
| Tabocas do Brejo Velho | 2020-06-20 | NA | NA |
| Tanhaçu | 2020-05-30 | 2020-04-25 | 2020-04-25 |
| Tanque Novo | 2020-06-05 | NA | NA |
| Tanquinho | 2020-05-30 | 2020-04-18 | 2020-06-24 |
| Taperoá | 2020-04-15 | 2020-05-08 | 2020-07-23 |
| Tapiramutá | 2020-06-25 | NA | NA |
| Teixeira de Freitas | 2020-03-24 | 2020-04-03 | 2020-04-14 |
| Teodoro Sampaio | 2020-06-10 | 2020-06-19 | 2020-06-19 |
| Teofilândia | 2020-05-14 | 2020-04-14 | 2020-06-22 |
| Teolândia | 2020-06-06 | NA | NA |
| Terra Nova | 2020-05-26 | 2020-03-31 | 2020-06-06 |
| Tremedal | 2020-06-05 | 2020-04-19 | NA |
| Tucano | 2020-05-06 | 2020-04-15 | 2020-06-19 |
| Uauá | 2020-05-30 | 2020-04-02 | NA |
| Ubaíra | 2020-06-06 | 2020-04-07 | 2020-04-19 |
| Ubaitaba | 2020-04-26 | 2020-04-03 | 2020-05-16 |
| Ubatã | 2020-04-09 | 2020-04-19 | 2020-04-23 |
| Uibaí | 2020-06-06 | 2020-04-21 | 2020-04-21 |
| Umburanas | 2020-05-09 | 2020-05-13 | NA |
| Una | 2020-04-14 | 2020-04-16 | 2020-04-16 |
| Urandi | 2020-05-18 | NA | NA |
| Uruçuca | 2020-04-04 | 2020-04-14 | 2020-04-18 |
| Utinga | 2020-04-03 | 2020-04-28 | NA |
| Valença | 2020-04-17 | 2020-04-01 | 2020-04-08 |
| Valente | 2020-04-15 | 2020-04-17 | NA |
| Várzea da Roça | 2020-05-14 | 2020-04-21 | NA |
| Várzea do Poço | 2020-07-20 | NA | NA |
| Várzea Nova | 2020-05-01 | NA | NA |
| Varzedo | 2020-05-22 | 2020-04-19 | 2020-07-03 |
| Vera Cruz | 2020-04-11 | 2020-04-02 | 2020-04-05 |
| Vereda | 2020-05-14 | NA | NA |
| Vitória da Conquista | 2020-04-01 | 2020-04-01 | 2020-04-06 |
| Wagner | 2020-06-30 | 2020-05-27 | NA |
| Wanderley | 2020-06-03 | NA | NA |
| Wenceslau Guimarães | 2020-05-20 | 2020-05-18 | 2020-05-18 |
| Xique-xique | 2020-05-22 | 2020-04-10 | NA |
